# Association between radiological parameters and clinical and molecular characteristics in human somatotropinomas

**DOI:** 10.1038/s41598-018-24260-y

**Published:** 2018-04-18

**Authors:** María R. Alhambra-Expósito, Alejandro Ibáñez-Costa, Paloma Moreno-Moreno, Esther Rivero-Cortés, Mari C. Vázquez-Borrego, Cristóbal Blanco-Acevedo, Álvaro Toledano-Delgado, María S. Lombardo-Galera, Juan A. Vallejo-Casas, Manuel D. Gahete, Justo P. Castaño, María A. Gálvez, Raúl M. Luque

**Affiliations:** 1 Maimonides Institute of Biomedical Research of Cordoba, Córdoba, 14004 Spain; 2 Reina Sofia University Hospital (HURS), Córdoba, 14004 Spain; 3 Service of Endocrinology and Nutrition, HURS, Córdoba, 14004 Spain; 4 Department of Cell Biology, Physiology and Immunology, Universidad de Córdoba, Córdoba, 14004 Spain; 5 CIBER Fisiopatología de la Obesidad y Nutrición (CIBERobn), Córdoba, 14004 Spain; 6 Campus de Excelencia Internacional Agroalimentario (ceiA3), Córdoba, 14004 Spain; 7 Service of Neurosurgery, HURS, Córdoba, 14004 Spain; 8 Radiology Service, HURS, Córdoba, 14004 Spain

## Abstract

Acromegaly is a rare but severe disease, originated in 95% of cases by a growth hormone-secreting adenoma (somatotropinoma) in the pituitary. Magnetic resonance imaging (MRI) is a non-invasive technique used for the diagnosis and prognosis of pituitary tumours. The aim of this study was to determine whether the use of T2-weighted signal intensity at MRI could help to improve the characterisation of somatotropinomas, by analysing its relationship with clinical/molecular features. An observational study was implemented in a cohort of 22 patients (mean age = 42.1 ± 17.2 years; 59% women; 95% size>10 mm). Suprasellar-extended somatotropinomas presented larger diameters vs. non-extended tumours. T2-imaging revealed that 59% of tumours were hyperintense and 41% isointense adenomas, wherein hyperintense were more invasive (according to Knosp-score) than isointense adenomas. A higher proportion of hyperintense somatotropinomas presented extrasellar-growth, suprasellar-growth and invasion of the cavernous sinus compared to isointense adenomas. Interestingly, somatostatin receptor-3 and dopamine receptor-5 (DRD5) expression levels were associated with extrasellar and/or suprasellar extension. Additionally, DRD5 was also higher in hyperintense adenomas and its expression was directly correlated with Knosp-score and with tumour diameter. Hence, T2-weighted MRI on somatotropinomas represents a potential tool to refine their diagnosis and prognosis, and could support the election of preoperative treatment, when required.

## Introduction

Acromegaly is a rare disease, with a prevalence of only 35–75 cases per million inhabitants, and an incidence of 3–4 cases per million inhabitants/year^1,2^. It is caused by over-production of growth hormone (GH), which in ~95% of cases is due to a pituitary adenoma (PA)^3^, usually presenting as a macroadenoma on diagnosis^4^. Magnetic resonance imaging (MRI) has greatly improved the success of clinical management in recent years and is now a key element in the diagnosis of patients with acromegaly. Thus, MRI is a non-invasive diagnostic test that can help to characterise GH-secreting adenomas and could aid to predict their clinical behavior. Specifically, some studies have suggested the existence of a relationship between the intensity of the adenoma on T2-weighted imaging and its characteristics^5^, pathology^5–7^ and response to somatostatin analogues^6,8^. However, these studies were conducted in small series of PAs and the findings are still inconsistent and/or inconclusive, which could in part be also due to the characteristics of GH-secreting adenomas (i.e. unlike other PA types that are usually hyperintense on T2-weighted imaging, GH-secreting adenomas can present varying intensities). Yet, the reason for this variability in T2 intensity is unclear^4^.

The behaviour and response to treatment of GH-secreting adenomas depend on their clinical, biochemical, imaging and genetic characteristics, which cannot still be used, routinely and reliably, to predict the aggressiveness of the tumour at the time of diagnosis. Tumour markers such as securin or pituitary tumour–transforming gene (PTTG1)^9^ and the Ki67 proliferation marker^10^ can be evaluated from the tumour, as well as several receptors of central and hypothalamic hormones such as somatostatin (sst), dopamine (DRD), ghrelin (GHSR1a) or vasopressin (AVPR1b) receptors^11–16^. Specifically, somatostatin receptors 2 (sst2) and 5 (sst5) predominate (90%-95%) in GH-secreting tumours^12,17^, and the expression of these sst subtypes could provide a predictor of response to somatostatin analogue (SSA) therapy^12,13,18^, which would be clinically relevant as a considerable number of patients are or become totally or partially resistant to SSA therapy^19–22^. Interestingly, in an attempt to clarify patient response to SSAs, various studies have shown that large tumours with low sst2 expression are generally: (1) poorly granulated, (2) exhibit low positivity for GH, and (3) more invasive. While densely granulated tumours with increased sst2 expression seem to respond better to SSA^23–27^.

Nevertheless, the molecular analysis of receptors is only possible after surgery, and it is not available in all hospitals. Thus, molecular parameters have not been developed hitherto to predict which patients will respond to treatment, or the progression of the disease. In this context, our main objective was to determine whether the T2-weighted intensity of the adenoma correlates to the clinical and pathological characteristics of the patient at diagnosis of acromegaly and/or the genetic expression of different tumour-related receptors, in order to more precisely classify the tumours and, if required, to predict the most effective medical preoperative treatment (which could be indicated in the case of extensive cavernous sinus invasion, absence of chiasmal compression, or poor surgical candidate^28^).

## Results

### Patients characteristics

Twenty-two patients were included in the study (41% men; 59% women). Baseline clinical and analytical characteristics of the patients are shown in Supplemental Table 1 and Supplemental Table 2, respectively. Mean age at diagnosis of acromegaly was 42.09 ± 17.15 years (42.56 ± 18.08 in men vs. 41.77 ± 17.22 in women; p = 0.919). Clinical manifestations at diagnosis of acromegaly did not differ between men and women, except for headache, which was more frequent in women. Interestingly, the prevalence of obstructive sleep apnoea syndrome (OSAS) is lower than expected according to the latest review^29^, but similar to some national acromegaly registries^30^. In this sense, it should be mentioned that, in our study, patients were classified as OSAS if the Lung Specialist recommended the use of continuous positive airway pressure (CPAP) (Supplemental Table 1). Circulating Insulin-like growth factor 1 (IGF-1) levels at diagnosis were 575.45 ± 287.51 ng/ml (605.84 ± 266.64 in men vs. 607.81 ± 280.94 in women; p = 0.987), thus presenting no differences between genders (Supplemental Table 2). In addition, although there is no consensus in the current bibliography^31–33^, our data indicate that in this cohort, circulating basal and nadir GH level following oral glucose tolerance test (OGTT) were both significantly higher in men than in women (Supplemental Table 2).

**Table 1:** Particular characteristics of GH-secreting adenomas stratified by maximum tumour diameter.

Tumour size	≤10 mm	10–20 mm	>20 mm	P value
Number	1	12	9	—
Sex (♂/♀)	0 vs. 1	6 vs. 7	3 vs. 5	0.551
Age at diagnosis	36	41.55 ± 17.74	41.13 ± 19.28	0.961
IGF-1 (nd/dL)	500.43	568.49 ± 276.66	626.34 ± 307.09	0.673
Basal GH (ng/dL)	5.05	7.27 ± 8.07	11.39 ± 9.80	0.537
Nadir GH (ng/dL)	5.53	6.37 ± 7.88	12.00 ± 11.36	0.311

GH-secreting adenomas were classified into 3 groups by maximum tumour diameter (less than 10 mm, between 10 and 20 mm, and greater than 20 mm) and demographic (sex and age at diagnosis) and clinical (levels of IGF-1 and basal and nadir GH after OGTT) parameters were evaluated.

**Table 2:** Characteristics of pituitary adenomas based on Knosp score.

**Knosp score**	0	1	2	3	4	P value
%	17.6%	17.6%	23.5%	11.8%	29.4%	—
Age (years)	35.67 ± 7.51	29.33 ± 14.50	29.50 ± 13.03	62.00 ± 15.56	47.40 ± 18.68	0.116
IGF-1 (ng/mL)	547.80 ± 116.25	423.18 ± 264.93	516.47 ± 300.18	493.05 ± 311.95	597.91 ± 401.64	0.956
Nadir GH (ng/mL)	11.41 ± 11.80	10.95 ± 9.32	9.79 ± 10.46	8.84 ± 7.98	8.75 ± 10.08	0.861
Sex (♂/♀)	3/1	1/3	3/3	0/2	2/4	0.067
APD (mm)	10.00 ± 4.58	15.20 ± 4.70	18.70 ± 3.50	16.90 ± 1.56	26.50 ± 5.54	**0.004**
TD (mm)	8.77 ± 2.85	17.87 ± 3.01	18.15 ± 4.57	17.65 ± 3.32	27.12 ± 9.36	**0.022**
CCD (mm)	11.10 ± 4.76	15.17 ± 4.76	16.97 ± 5.98	17.90 ± 4.38	29.60 ± 6.42	**0.003**

APD: anteroposterior diameter; TD: transverse diameter; CCD: craniocaudal diameter.

### Magnetic Resonance Imaging (MRI)

Nearly all patients (95%) presented macroadenoma when examined by MRI. Mean anteroposterior, transverse and craniocaudal diameter (APD, TD and CCD, respectively) were 18.62 ± 6.78, 18.97 ± 8.09 and 18.87 ± 8.28 mm, respectively. Interestingly, the classification of adenomas by size into 3 groups, less than 10 mm, between 10 and 20 mm, and greater than 20 mm, as previously reported^4^ revealed that patients with larger adenomas had numerically higher levels of IGF-1 and basal or nadir GH after OGTT, although these changes were not statistically significant within groups (Table 1).

**Figure 1:**
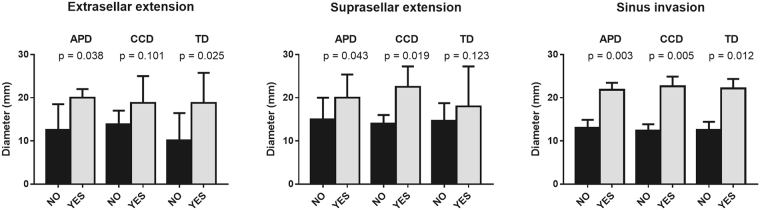
Association between extrasellar/suprasellar extension and invasion and the diameter of somatotropinomas. The diameter of GH secreting adenomas was measured in millimetres. APD: anteroposterior diameter; CCD: craniocaudal diameter; TD: transverse diameter. Data represent median ± interquartile range in extrasellar/suprasellar extension and mean ± SEM in invasion graphs. Differences were evaluated by Student t or Mann-Whitney U tests depending on the normality of each data distribution.

In total, 79% of patients presented extrasellar tumour extension, wherein 63% exhibited suprasellar growth. As expected, tumours with extrasellar/suprasellar growth and those that invaded the sinuses were larger than the tumours without extension and/or invasion (Fig. 1). No differences in tumour size was found in terms of sex, age at diagnosis, IGF-1 level, and basal or nadir GH after OGTT.

Additionally, right cavernous sinus space invasion was observed in 26% of patients, left sinus invasion in 21%, and invasion of both spaces in 16%. A higher Knosp score was associated with larger tumour diameter (Table 2), but not with any other study parameter, including IGF-1 levels.

**Figure 2:**
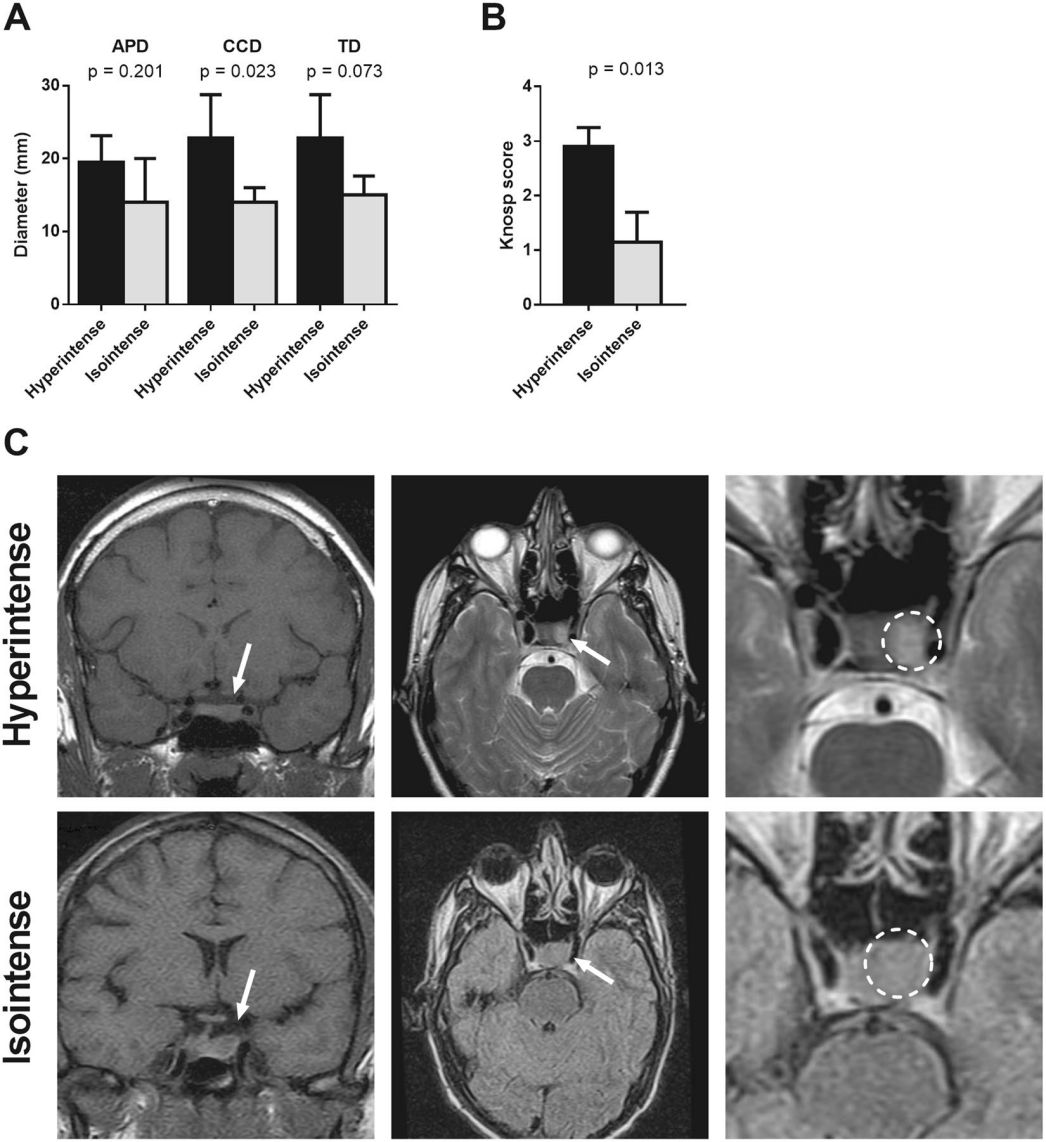
Association between T2-signal and the diameter (**A**) and Knosp score (**B**) of somatotropinomas. The diameter of GH secreting adenomas was measured in millimetres. APD: anteroposterior diameter; TD: transverse diameter; CCD: craniocaudal diameter. Data represent median ± interquartile range. (**C**) Classification of T2-weighted signal of GH secreting pituitary adenomas: T2-hyperintense adenomas, upper-panel and T2-isointense adenomas, lower-panel. Tumour region was indicated with an arrow in coronal MRI (left), and in T2-weighted axial MRI (middle), the tumour area was indicated by a circle in right panel.

Finally, T2 imaging indicated that 59% of adenomas were hyperintense and 41% isointense. No hypointense adenomas were found in our series. T2 intensity (hyperintense vs. isointense groups) did not differ significantly when compared with sex, age, IGF-1 levels, or nadir GH at diagnosis. On T2-weighted MRI, CCD and TD of hyperintense adenomas were, or tended to be, greater than isointense tumours (p = 0.023 and p = 0.073, respectively) (Fig. 2A). Moreover, mean Knosp score for hyperintense adenomas was higher than in isointense tumours (Fig. 2B). Representative images of hyperintense and isointense adenomas are shown in Fig. 2C.

All hyperintense adenomas presented extrasellar growth vs. only 43% of isointense adenomas (p = 0.023). Furthermore, tumours that presented suprasellar growth or those that invaded the cavernous sinus space were more commonly hyperintense compared to isointense adenomas (in both cases, 90% vs. 42.9%; p = 0.036). Specifically, in the case of hyperintense adenomas, 10% were only invasive, 10% were only suprasellar, and 80% were both. However, no statistically significant differences were found between hyperintense and isointense tumours with regard to treatment or disease control, as specified in Methods section (Table 3).

**Table 3:** Treatment overview of the patients according to T2-intensity.

Pharmacological Treatment	Number (%)			Radiotherapy			Disease control (%)		
	Total	Iso	Hyper	Total	Iso	Hyper	Total	Iso	Hyper
No treatment	5 (22.7)	1	4	0	0	0	5	1	4
Oct	2 (9.1)	1	1	1	1	0	0	0	0
Lan	5 (22.7)	3	2	3	2	1	2	1	1
Cab	3 (13.6)	2	1	0	0	0	3	2	1
Oct + Cab	2 (9.1)	0	2	0	0	0	0	0	0
Lan + Cab	3 (13.6)	1	2	1	1	0	3	1	2
Pas + Cab	1 (4.5)	1	0	1	1	0	1	1	0
Peg + Cab	1 (4.5)	0	1	1	0	1	0	0	0
Total	22 (100)	9	13	7	5	2	14 (63.7)	6	8

Iso: isointense adenomas; Hyper: hyperintense adenomas; Oct: octreotide; Lan: lanreotide; Cab: cabergoline; Peg: pegvisomant.

### Molecular analysis of the pituitary adenomas

**Figure 3:**
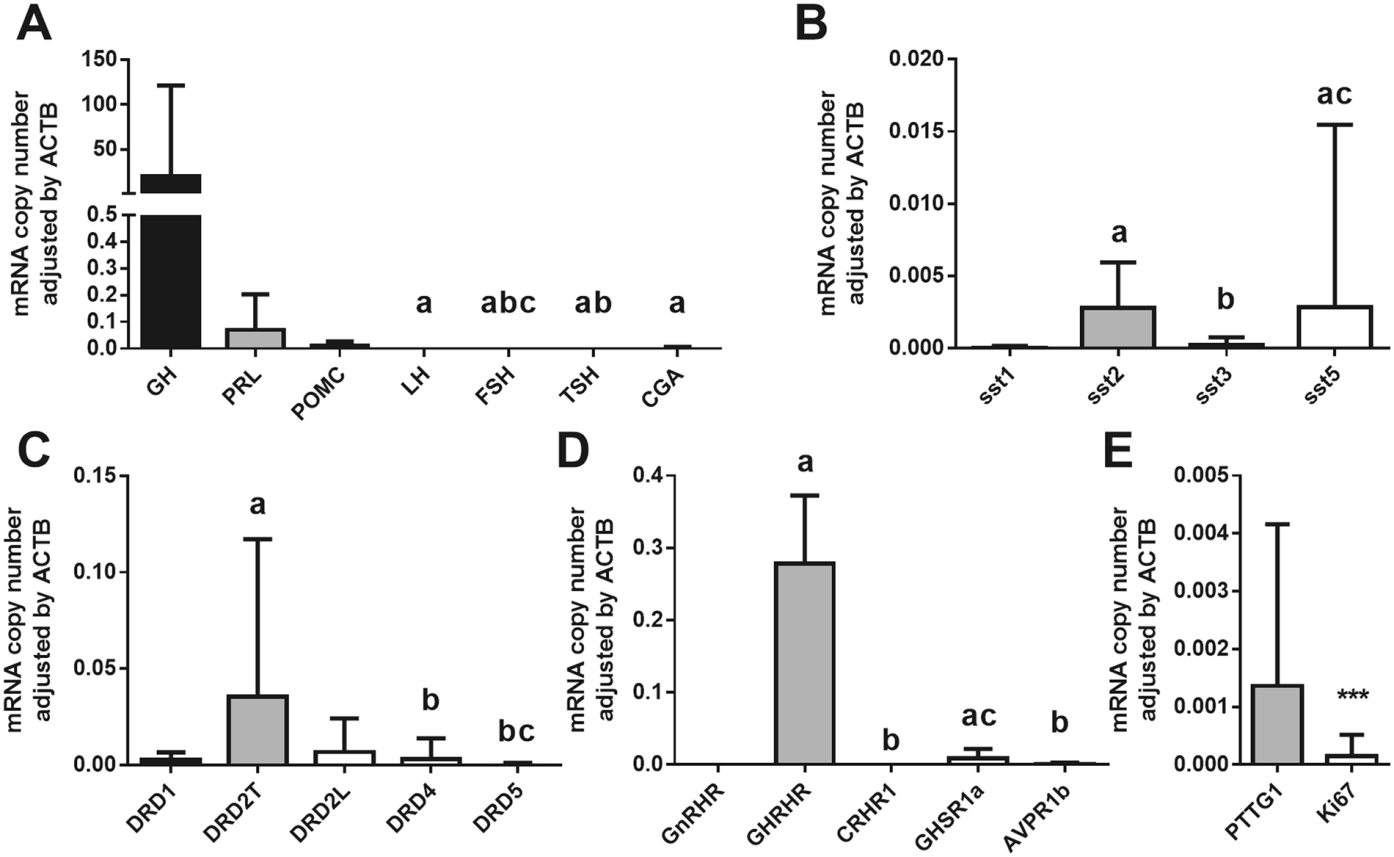
Molecular characterisation of the somatotropinomas. Seventeen somatotropinomas were available to determine by quantitave real-time PCR (qPCR) the expression profile of: (**A**) pituitary hormones [growth hormone (GH), prolactin (PRL), proopiomelanocortin (POMC), luteinizing hormone (LH), follicle stimulating hormone (FSH), thyroid-stimulating hormone (TSH) and the alpha subunit of the glycoproteins (CGA)]; (**B**) the five somatostatin receptors subtypes (sst1-5); (**C**) the five dopamine receptors subtypes (DRD1-5), including the total and large isoform of DRD2 (DRD2T and DRD2L, respectively); (**D**) other receptors [gonadotropin releasing hormone receptor (GnRHR), growth hormone releasing hormone receptor (GHRHR), corticotropin releasing hormone receptor (CRHR1), ghrelin receptor (GHSR1a), arginine-vasopressin receptor 1b (AVPR1b); and (**E**) and two proliferation markers [securin (PTTG1) and Ki67]. Data represent median ± interquartile range of absolute expression levels (copy number) of each transcript adjusted by the expression levels of a control gene (ACTB). Values in A-D that do not share a common letter are statistically different (P < 0.05) using a Kruskal-Wallis test followed by a Dunn’s multiple comparisons test. Mann-Whitney test was used for E (*** indicates P < 0.001).

The analysis of the molecular profile (Fig. 3A) confirmed that somatotropinomas expressed high levels of GH (19.586 ± 113.743 copies/adjusted by ACTB) and low levels of other hormones, being PRL (0.07 ± 0.147 copies/adjusted by ACTB) and POMC (0.01 ± 0.017 copies/adjusted by ACTB) the most expressed among them.

Somatostatin and dopamine receptor subtypes were differently expressed (Fig. 3B,C). Particularly, sst5 and sst2 were the most frequently expressed (95% of the tumours in both cases), followed by sst3 (74%) and sst1 (59%). In the case of dopamine system, DRD2 was most frequently expressed receptor (100% of the cases), followed by DRD4 (89%), DRD1 (87%) and DRD5 (80.0%). As expected, GH-releasing hormone receptor (GHRHR) and ghrelin receptor (GHSR1a) were also expressed at high levels in these somatotropinomas (100% and 92% of the cases, respectively; Fig. 3D), being GHRH-R mRNA levels significantly higher than those of GHSR1a. In contrast, other receptors subtypes involved in the function of different pituitary cell types, such as gonadotropin-releasing hormone receptor (GnRHR), corticotropin-releasing hormone receptor (CRHR1) or vasopressin receptor (AVPR1b) were not noticeably expressed in somatotropinoma samples (Fig. 3D). We also measured two well-known proliferation markers in our cohort of somatotropinoma samples, and found that PTTG1 mRNA levels were higher than Ki67 levels (Fig. 3E).

**Figure 4:**
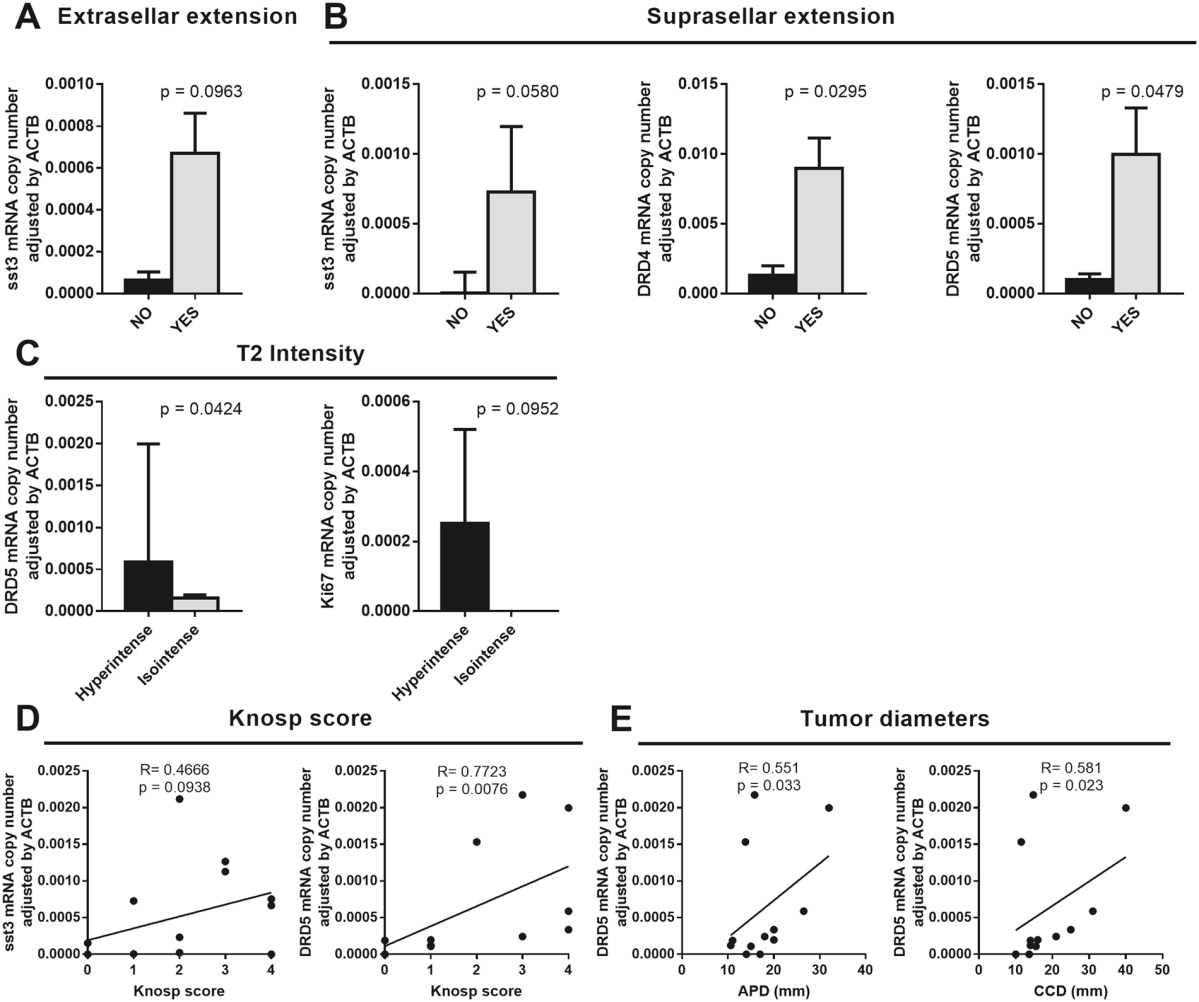
Association between molecular markers and tumour phenotype. (**A** and **B**) Association between the presence of extrasellar and suprasellar extension and mRNA levels of sst3, DRD4 and DRD5. (**C**) Association between T2 signal and mRNA levels of DRD5 and Ki67. (**D** and **E**) Correlation study between Knosp score and tumour diameter and mRNA level of sst3 and DRD5.

Interestingly, adenomas with extrasellar and suprasellar extension tended to present higher sst3 levels (Fig. 4A,B). In the same line, adenomas with suprasellar extension presented higher levels of DRD4 and DRD5 than adenomas without suprasellar extension (Fig. 4B). No differences were found concerning the invasion of cavernous sinuses. Moreover, compared to isointense adenomas, hyperintense adenomas at T2 imaging presented higher expression levels of DRD5, and a trend for enhanced Ki67 expression (Fig. 4C). Nevertheless, no other receptors or hormones were different in hyperintense vs. isointense adenomas at mRNA level. Additionally, a direct correlation was also found between expression of DRD5 and the Knosp score, which also showed a trend to associate with the expression of sst3 (Fig. 4D). Finally, DRD5 expression was directly correlated with both APD and CCD (Fig. 4E).

## Discussion

The main objective of this study was to determine whether the T2-weighted intensity of the adenoma correlates to the patient’s clinical and pathological characteristics at diagnosis of acromegaly, or to the genetic expression of different tumour-relevant receptors and tumour-related markers. To that end, we retrospectively analysed a series of 22 patients bearing a GH-producing tumour, in whom T2-weighted intensity analysis of the adenoma were available. Interestingly, we found a predominance of hyperintense adenomas in our series (59%), and no evidence of hypointense tumours, even though these latter have been previously reported to account for between 27% and 50% of all adenomas^4–8^. This discrepancy is probably related to differences in the definition of adenoma intensity on T2-weighted MRI among published studies. Our approach to intensity definition is similar to that used by Potorac *et al*., who compared T2-weighted signal intensity of adenomas with that of normal pituitary tissue, and on this basis classified them as hypo-, iso-, or hyper-intense^4^. When normal tissue was concealed, signal intensity was compared with that of temporal lobe grey matter. In other studies, signal intensity of adenomas on T2-weighted MRI was classified as hyperintense when higher than grey matter intensity, hypointense when lower than white matter intensity, and isointense when higher than white matter intensity but lower than grey matter intensity^5,6,8,34,35^. However, it seems reasonable to conceive that studies involving MRI evaluation of pituitary tumours should compare T2-weighted signal intensity of adenoma tissue with that of normal pituitary tissue whenever possible, as we have performed in this study.

In our series, hyperintense adenomas tended to be larger than isointense tumours, especially if APD is considered, and more invasive, presenting a higher Knosp score. In fact, all hyperintense adenomas presented extrasellar growth, wherein 90% of them were suprasellar. Invasion of the cavernous sinus space was also more common in hyperintense vs. isointense adenomas. These findings are consistent with those of other published reports^4,6,8^, and suggest that adenomas that are hyperintense on T2-weighted MRI are potentially more proliferative than non-hyperintense tumours. Additionally, Heck *et al*., reported that adenomas that are hyperintense on T2-weighted MRI tend to be larger but secrete less GH and hence are associated with lower IGF-1 levels, which could be explained by the finding that all sparsely granulated adenomas studied were hyperintense, while the densely granulated ones were mainly hypo or isointense^6,8^. In this sense, it has been reported that sparsely granulated somatotropinomas are typically larger, more invasive, less responsive to treatment and more frequently found in younger patients, compared to densely granulated tumours^36^, which indicates that hyperintense adenomas (which usually correspond to sparsely granulated adenomas) may be more aggressive. Additionally, it has also been reported that hyperintense adenomas differed significantly in GH and IGF-1 levels from the hypo- and isointense group^37^, wherein the absolute IGF-1 values tended to be lower in the hyperintense group despite a trend towards younger age. Moreover, as tumour size tended to be larger in the hyperintense group, the amount of GH secreted respective to the tumour volume, also known as *GH-index*, was lower in the hyperintense adenomas and, furthermore, the response to an octreotide test was blunted in these patients^37^. Remarkably, a similar phenotype has been reported in sparsely-granulated adenomas, wherein a trend towards lower *GH-index* and blunted response to octreotide test dose has been described^27,38^. Therefore, these data indicate that the hypo and hyperintense tumours might represent biologically different subgroups with a divergent secretory behaviour. Further supporting this idea, recent guidelines for acromegaly management have also noted the potential utility of using T2 intensity to optimize patient management^28^.

Few studies have explored to date the potential association between MRI features of pituitary adenomas and their molecular profile. In our series of somatotropinomas, the overall pattern of gene expression appeared to be in line with that reported in comparable previous studies. Thus, specifically, GH was the most abundantly expressed hormone, followed by PRL and POMC^17^. Similarly, sst5 and sst2, and DRD2 were the mainly expressed somatostatin and dopamine receptors in tumoural somatotropes^12,16,17,39,40^. As previously reported, the most expressed receptors for hypothalamic regulators were those for GHRH^41^ and ghrelin^14,42–46^, while the expression of receptors for other hypothalamic modulators such as GnRHR, AVPR1b and CRHR1 are virtually lacking, as previously observed^47^. A recent study has evaluated the relation between T2 intensity and sst2 and sst5 immunoreactivity, and, interestingly, the level of sst5, but not sst2, was inversely correlated with T2 intensity^35^. In our series, no apparent associations were found between sst2 or sst5 expression and T2 intensity. However, the molecular study revealed, for the first time, that although the expression of sst3, DRD4 and DRD5 is low in these samples, it is increased in adenomas with suprasellar extension compared with the tumours with no suprasellar growth. Of note, these receptors are not currently targeted with specific pharmacological therapies for pituitary adenomas, as there are no available selective agonists for these receptors. Our present results suggest that this possibility is worth to be explored, due to its putative therapeutic implications, and, therefore, further studies should be implemented in this regard. Moreover, we found that T2 hyperintense tumours presented an increased expression of DRD5 and Ki67 at mRNA level, together with the larger size and invasive nature, lending credence to the notion that DRD5 might be a marker of poor prognosis. In support to this contention is the direct correlation between DRD5 and APD and CCD diameter, which is also further reinforced by the direct association between Knosp score and DRD5 and sst3 expression. These findings, which are reported for the first time in the literature, suggest that a combined analysis of T2 intensity and expression of these molecular markers could help to guide therapeutic choices at the time of diagnosis. Indeed, our results could suggest that larger tumours, characterised by poorer prognosis for surgical resolution and bearing a higher expression of DRD5 and sst3, could be possibly treated before surgery using alternative DRD agonists (i.e. with high affinity for DRD5) or pasireotide (a multireceptor ligand with affinity for sst1, sst2, sst3 and sst5), insofar the mRNA of these receptors is correctly transcribed to protein, as previously reported in neuroendocrine tumours^48^.

In conclusion, in this study we found that on T2-weighted imaging, hyperintense GH-secreting pituitary adenomas are larger and tend to be more aggressive due to the invasion of adjacent structures, and these features may be linked to the expression of specific molecular markers. Hence, detailed preoperative imaging studies might help to predict the ideal preoperative treatment and management in these patients. However, further studies in larger cohorts of patients using the same criteria for defining pituitary adenoma characteristics on MRI, collecting the pharmacological and/or radiotherapy treatment outcome and analysing the molecular profile are required to clarify the implications of MRI in somatotropinoma characterization and in the choice of medical treatment, if required.

## Methods

### Patients

This is a retrospective, single-centre study (Reina Sofía University Hospital in Cordoba, Spain) including all patients diagnosed with acromegaly that had a preliminary MRI of the pituitary prior to the start of treatment, and with a molecular expression profile of the tumour immediately after surgery. Only patients with a confirmed diagnosis of acromegaly were included. This study was approved by the Hospital Ethic Committee and conducted in accordance with the principles of the Declaration of Helsinki. Written informed consent was obtained from all patients.

Diagnosis of acromegaly was based on the presence of classic clinical features and on a result of GH ≥1 ng/dl in a 75 mg OGTT, elevated IGF-1 levels according to the patient’s reference range for age and sex and/or on the presence of a pituitary adenoma by MRI. In all cases, histopathology of the surgical specimen confirmed the presence of a GH-producing adenoma. Criteria to consider successful resolution of acromegaly (cured patients) are disappearance of the pituitary adenoma, normalisation of GH and IGF-1 levels, and suppression of GH levels following OGTT (below 1 or 0.4 ng/l), without the need for medical treatment. Additionally, acromegaly controlled with medication was defined as GH and IGF-1 levels within normal ranges in a patient under treatment^49^. Demographic variables such as age, sex and race were collected, together with clinical characteristics and results of different tests at diagnosis. Exclusion criteria were pregnancy or breastfeeding and refusal to sign the informed consent form.

### Magnetic Resonance Imaging

Imaging studies were performed using high resolution 1.5 T MRI instrument (MAGNETOM Aera 1.5 Tesla, Siemens). To qualify for inclusion in the study, patients must have at least 1 coronal and 1 sagittal T1- and T2-weighted sequence. MRI images were independently analyzed by two neuro-radiologists expert in the interpretation of MRI pituitary images, wherein their evaluations were 100% concurrent. The following parameters were recorded: anteroposterior diameter (APD), transverse diameter (TD) and craniocaudal diameter (CCD), extrasellar growth, presence or absence of suprasellar contact with optic chiasm, invasion of cavernous sinus space, and T1 and T2 signal intensity. T2-weighted intensity was classified as hypo-, iso- or hyper-intense compared with normal pituitary tissue (n = 17/22 cases). When the pituitary tissue was hidden, as in the case of pituitary macroadenomas, the intensity was compared with that of temporal lobe grey matter (n = 5/22 cases), as previously reported^4^. Invasion of cavernous sinus spaces was classified according to the system of grading invasion of cavernous sinus by pituitary adenomas (grade 0–4) developed by Knosp *et al*.^50^

### Hormonal analysis

Absolute IGF-1 levels at diagnosis were determined by an immunoradiometric assay (#A15729 Immunotech SAS, Marseille, France) and compared to the upper limit of normal adjusted by age and sex. To avoid ambiguity, IGF-1 levels are expressed as a standard deviation score with respect to the reference range for age, sex, pubertal stage, and determination method. GH was determined by chemiluminescence by immunoradiometric assay (#HGH-RIACT, Cisbio Bioassays, Codolet, France). All the information regarding specificity, detectability, and reproducibility for each of the assays can be accessed at the website of the companies.

### Specimen analysis

GH-secreting pituitary adenomas tissue samples were obtained during transsphenoidal surgery. The fact that the tissue piece collected by our laboratory corresponded to somatotropinoma tissue was confirmed by 3 separate methods, as previously reported^15^: by the examination of an anatomopathologist expert in pituitary, by testing of the hormonal phenotype using cell-blotting assays, and by assessing the molecular screening using qPCR evaluating the genes described above, which has been repeatedly proven in our laboratory^11^. Subsequently, a portion of the tumour was retained for pathological examination and the remaining fragments were snap frozen in liquid nitrogen until RNA extraction. Total RNA was isolated with the AllPrep DNA/RNA/Protein Mini Kit with deoxyribonuclease treatment using RNase-Free DNase Set (Qiagen, Limburg, Netherlands), following the instructions of the manufacturer. In all cases, total RNA concentration and purity was assessed using Nanodrop 2000 spectrophotometer (Thermo Scientific). Total RNA was retro-transcribed using random hexamer primers and the cDNA First Strand Synthesis kit (Thermo Scientific). Details regarding the quantitative PCR (qPCR) procedure used to determine the absolute expression levels of the different transcripts included in this study have been previously reported by our laboratory^11–15^. Specifically, the genes analysed in this study were: growth hormone (GH), prolactin (PRL), proopiomelanocortin (POMC), follicle stimulating hormone (FSH), luteinising hormone (LH), thyroid-stimulating hormone (TSH) and the alpha subunit of the glycoproteins (CGA); somatostatin receptor subtypes (sst1, sst2, sst3, sst5), dopamine receptor subtypes (DRD1, DRD2T, DRD2L, DRD4, DRD5), other hormonal receptors [gonadotropin releasing hormone receptor (GnRHR), growth hormone releasing hormone receptor (GHRHR), corticotropin releasing hormone receptor (CRHR1), ghrelin receptor (GHSR1a) and arginine-vasopressin receptor 1b (AVPR1b)] and two proliferation markers (Ki67 and PTTG1). Specific sets of primers for these genes have been previously validated and reported by our group^11^. To control for variations in the amount of RNA used in the retro-transcription reaction and the efficiency of the retro-transcription reaction, mRNA copy numbers of the different transcripts analysed were adjusted by beta-actin (ACTB) expression (used as housekeeping gene, as recently reported^39^).

### Statistical analysis

The descriptive analysis of qualitative variables in each category was expressed in terms of absolute frequencies and percentage. Quantitative variables are expressed as mean ± standard variation, using the Shapiro-Wilk analysis to test for normality. mRNA data adjusted by ACTB expression were expressed as median ± interquartile range. Wherever non-normal variables were detected, the corresponding non-parametric test was used. The chi-square test was used to associate qualitative variables. Means were compared using the Student’s t test for parametric variables, and the Mann-Whitney U test for non-parametric variables. In the case of multiple comparison, a Kruskal-Wallis test followed by a Dunn’s multiple comparisons test was performed. Spearman’s correlation coefficient was used as a measure of correlation between non-parametric variables. Statistical significance was set at 5%, and the statistical analysis was performed using SPSS version 22.0 for Windows, and GraphPad Prism 7.0.

## Electronic supplementary material

Supplemental data
