# Supplementary material for: Association between radiological parameters and clinical and molecular characteristics in human somatotropinomas
Source: Sci Rep. 2018 Apr 18;8:6173. doi: 10.1038/s41598-018-24260-y (PMC5906631; doi:10.1038/s41598-018-24260-y)
Supplement: Supplementary file 1 — Supplemental data [file 41598_2018_24260_MOESM1_ESM.docx]

**Association between radiological parameters and clinical and molecular characteristics in human somatotropinomas**

**María R. Alhambra-Expósito, Alejandro Ibáñez-Costa, Paloma Moreno-Moreno, Esther Rivero-Cortés, Mari C. Vázquez-Borrego, Cristóbal Blanco-Acevedo, Álvaro Toledano-Delgado, María S. Lombardo-Galera, Juan A. Vallejo-Casas, Manuel D. Gahete, Justo P. Castaño, María A. Gálvez, Raúl M. Luque.**

**Supplemental Data**

**Supplemental Table 1.** **Signs and symptoms at diagnosis of acromegaly**.

|  | Total | Men | Women | P value |
| --- | --- | --- | --- | --- |
| Growth of extremities | 63.6% (14) | 66.7% (6) | 61.5% (8) | 0.584 |
| Hyperhidrosis | 36.4% (8) | 22.2% (2) | 46.2% (6) | 0.246 |
| OSAS | 13.6% (3) | 22.2% (2) | 7.7% (1) | 0.358 |
| Arthralgia | 36.4% (8) | 22.2% (2) | 46.2% (6) | 0.246 |
| Prognathism | 35.4% (8) | 55.6% (5) | 23.1% (3) | 0.135 |
| Decreased libido | 4.5% (1) | 11.1% (1) | 0.0% (0) | 0.409 |
| Altered menstrual cycle | 18.2% (4) | - | 33.3% (4) | - |
| Diabetes Mellitus | 22.7% (5) | 11.1% (1) | 30.8% (4) | 0.293 |
| Hypertension | 27.3% (6) | 22.2% (2) | 30.8% (4) | 0.523 |
| Osteoporosis | 13.6% (3) | 11.1% (1) | 15.4% (2) | 0.731 |
| Headache | 40.9% (9) | 11.1% (1) | 61.5% (8) | **0.025** |
| Visual disturbances:   - Quadrantanopia - Hemianopsia | 18.1% (4)  13.6% (3)  4.5% (1) | 11.1% (1)  11.1% (1)  0.0% (0) | 23.1% (3)  15.4% (2)  7.7% (1) | 0.450 |

Twenty-two patients were included in the study, 13 men and 9 women. OSAS: obstructive sleep apnoea syndrome. Data are expressed as percentage of cases (frequency).

**Supplemental Table 2.** **Analytical characteristics at diagnosis of acromegaly**.

|  | Total | Men | Women | P value |
| --- | --- | --- | --- | --- |
| Glucose (mg/dL) | 114.29 ± 45.55 | 119.22 ± 66.48 | 110.58 ± 22.88 | 0.508 |
| HbA1c (%) | 6.34 ± 0.97 | 6.46 ± 1.44 | 6.26 ± 0.59 | 1.000 |
| Total cholesterol (mg/dL) | 212.06 ± 46.70 | 201.13 ± 51.47 | 220.80 ± 43.22 | 0.274 |
| LDL (mg/dL) | 137.67 ± 47.86 | 132.13 ± 45.95 | 142.10 ± 51.34 | 0.633 |
| HDL (mg/dL) | 48.44 ± 17.85 | 42.00±11.80 | 53.60 ± 20.67 | 0.122 |
| Triglycerides (mg/dL) | 125.06 ± 56.88 | 126.63 ± 59.97 | 123.80 ± 57.55 | 0.965 |
| Prolactin (mcg/L) | 64.34 ± 99.82 | 45.15 ±40.75 | 77.14 ± 125.33 | 0.970 |
| GH (ng/ml) | 8.24 ± 8.25 | 11.19 ± 10.25 | 5.83 ± 5.58 | **0.010** |
| IGF-1 (ng/mL) | 575.45 ± 287.51 | 605.84 ± 266.64 | 554.40 ± 309.98 | 0.690 |
| Nadir GH after OGTT (ng/mL) | 7.45 ± 8.69 | 11.07 ± 3.83 | 3.83 ± 2.89 | **<0.001** |

Twenty-two patients were included in the study, 13 men and 9 women. GH: growth hormone; HbA1c: glycated haemoglobin; HDL: high-density lipoprotein; IGF-1: insulin-like growth factor; LDL: low density lipoprotein; OGTT: oral glucose tolerance test. Data are shown as Mean ± Standard Deviation.
